# Where is the Evidence? A Narrative Literature Review of the Treatment Modalities for Autism Spectrum Disorders

**DOI:** 10.7759/cureus.3901

**Published:** 2019-01-16

**Authors:** Srinivas Medavarapu, Lakshmi Lavanya Marella, Aneela Sangem, Ram Kairam

**Affiliations:** 1 Neurology, The Icahn School of Medicine at Mount Sinai, New York, USA; 2 Family Medicine, Medstar Shah Medical Group, Fort Washington, USA; 3 Pediatrics, Bronx Care Hospital, New York, USA; 4 Pediatrics, Developmental Neurology Associates, New York, USA

**Keywords:** evidence-based treatments for autism, applied behavioral analysis, autism spectrum disorders, pervasive developmental disorders, evidence-based interventions, treatment options for autism

## Abstract

The most important thing about autism spectrum disorder (ASD) is that there is, in fact, no cure for this disorder; however, currently, there are many claims of pharmacological and dietary therapies and behavioral interventions that are said to improve outcome or even lead to “cure” or “recovery.” It continues to remain a challenging condition for children and their families. Research conducted on many of these treatment modalities is limited and, consequently, sufficient evidence does not exist to support their use. The primary aim of this paper was to search for the evidence of the efficacy of each treatment for autism till now. We reviewed different treatment modalities and randomized clinical trials on each treatment to look for the evidence. Although there are interventions that may be effective in alleviating some symptoms and improving skills that help autistic persons lead more productive lives, proven benefits were observed only with applied behavioral analysis (ABA) and some psychopharmacologic agents.

## Introduction and background

Autism spectrum disorder (ASD) is a biologically based neurodevelopmental disorder characterized by impairments in two major domains: 1) deficits in social communication and social interaction and 2) restricted repetitive patterns of behavior, interests, and activities. ASD encompasses disorders previously known as autistic disorder (classic autism, sometimes called early infantile autism, childhood autism, or Kanner's autism), childhood disintegrative disorder, pervasive developmental disorder-not otherwise specified, and Asperger disorder (also known as Asperger syndrome).

According to the Centers for Disease Control and Prevention (CDC), ASD is a common disorder affecting 1 in 68 children, 1 in 42 boys, and is typically first recognized in early childhood [1]. Scientists are uncertain about the etiology of autism, but it is generally accepted that ASD is a multifactorial disorder, in which both genetics and the environment play a synergistic role.

ASD is a lifelong disorder that not only affects the patient with the condition but also their loved ones who must find ways to cope with the disorder. As a lifelong disorder with behavioral impairments, parents of children with autism tend to become frustrated and distressed by the lack of evidence-based treatment and medication available for treatment. This forces parents to become desperate for any intervention that will improve their child’s condition. Families of autistic children commonly turn to unproven alternative therapies that claim to be effective. There has been much controversy regarding the choice of these treatments for ASDs.

The creators of many treatments, both new and established, make impressive claims that are not supported by any form of controlled research. Furthermore, there are many educational professionals, who work with autistic individuals, who are reluctant to inform patients and their families about these “pseudoscientific” practices. Consequently, this places a huge burden on the families of autistic children, who are desperate for some kind of miraculous intervention. The families, who most often are not educated in research conducted in the field of autistic treatments, are easily misled. As a result, these families spend valuable time and money on unproven therapies rather than focusing their resources on therapies backed by extensive research, which have been proven to help autistic children. 

The most important thing to note about ASD is that there is, in fact, no cure for this disorder.

Materials and methods

**Figure 1:**
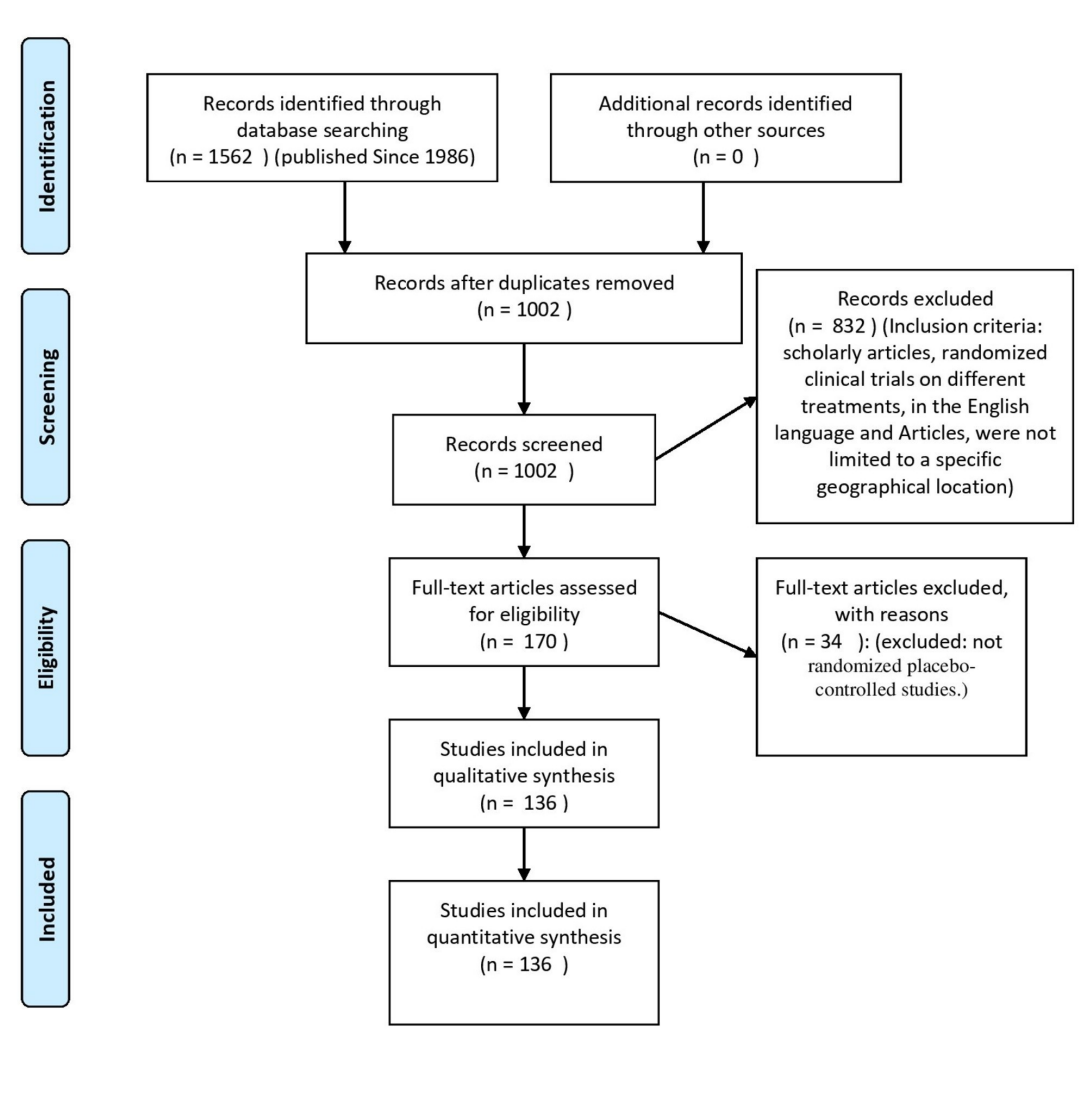
Study selection process flow diagram.

The article and data collection for this study was done using research journals and databases such as PubMed, Google Scholar, BioMed Central, EBSCO, Scopus, Web of Science, and MEDLINE. The reviewed articles were published from 1986 to the present time (Figure 1). During the search process, the following key phrases were used: autism spectrum disorder treatments, evidence-based treatments for autism, clinical trials on treatment of autism, and randomized control trials. Approximately 1500 articles were published between 1986 and 2018. The inclusion criteria were as follows: scholarly articles, randomized clinical trials on different treatments, articles and data were in English language, and articles were not limited to a specific geographical location. Approximately 130 articles were selected and carefully examined based on the relevance of their content to the topic of our study. Some of the references of these articles were also used in our work based on their relevance to our topic. These articles included different types of clinical trials like randomized trials, double-blind trials, open-label pilot studies, and randomized placebo-controlled studies. The following journals were used: Journal of Autism Developmental Disorders, Journal of Pediatrics, The New England Journal of Medicine, Journal of Psychopharmacology, Journal of Child Neurology, and American Journal of Psychiatry.

## Review

**Figure 2:**
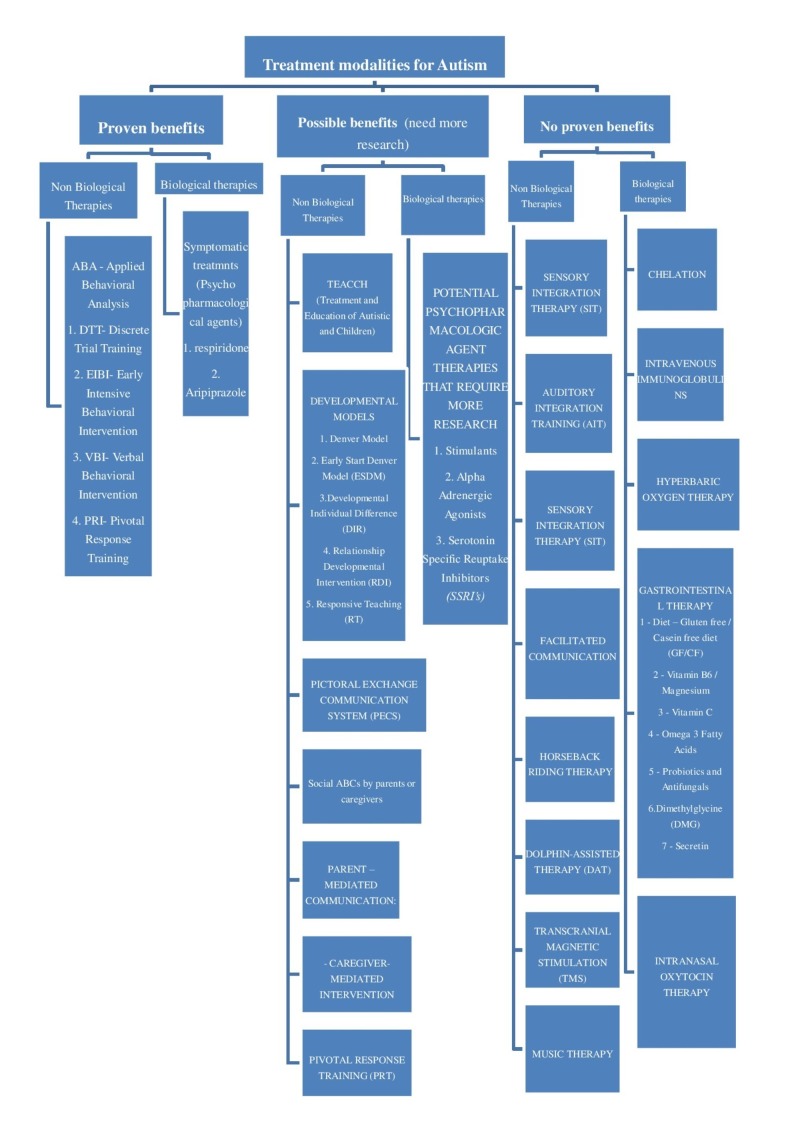
Various biological and non-biological therapies for autism. Categories of various treatment modalities with proven and unproven benefits.

Various biological and non-biological therapies that have claimed to improve outcome but have no proven benefits were analyzed in this review (Figure 2).

Biological therapies with no proven benefits

Chelation

Chelation is the process of eliminating harmful minerals, such as mercury, from the body. This type of therapy works with agents such as 2, 3 dimercaptosuccinic acid (DMSA) or 2, 3 dimercaptopropane-1-sulfonate (DMPS), which are administered to the individual. These agents bind to heavy metals and facilitate elimination.

Chelation therapy has become a popular intervention as a potential treatment for ASD. There have been suggestions of an association of autistic symptoms with environmental events and exposures. An example of such an association is with thiomerosal, an ethylmercury derivative, which is used as a preservative. Numerous studies done have concluded that there is no relationship between thiomerosal-containing vaccines and ASD [2].

Intravenous Immunoglobulins 

Intravenous immunoglobulins (IVIG) consist of pooled antibodies separated from the plasma of multiple donors. The theory behind the use of IVIG is that there is a relationship between the development of the fetal brain and the prenatal immune response. A clinical trial concluded that the use of IVIG to treat children with autism should be undertaken with great caution and only under formal research protocols [3]. The study included 10 children - five had no detectable change in behavior, four had mild improvement in hyperactivity and attention span, but parents of the children felt that this change was insufficient to warrant continuation of the therapy. There was a significant improvement in only one child who reverted to his autistic state in time. Another clinical trial that evaluated the use of IVIG as an autism treatment was disappointing [4]. In 2006, the American Academy of Allergy, Asthma and Immunology examined the evidence and concluded that the treatment was “unlikely to be beneficial” for autism.

*Hyperbaric Oxygen Therapy* 

Hyperbaric oxygen therapy (HBOT) is the medical use of oxygen at a level that is higher than atmospheric pressure. HBOT has been evaluated in some disorders of the central nervous system because of a related effect of increasing blood flow and or oxygen to the brain and decreasing inflammation. Since there have been hypotheses regarding brain and gut inflammation, brain hypoperfusion, and aberrant oxidative stress in ASD, there is increasing interest in using it [5]. An open clinical trial with 18 children with autism reported subjective improvements in several domains of autism [5]. Definite conclusions could not be made since it was an open-label study; it should, therefore, be interpreted with caution. A multicenter randomized, double-blind controlled trial was conducted to assess the efficacy of HBOT in children with autism [6]. The study concluded that the treatment group had significant improvements in overall functioning, social interaction, eye contact, receptive language, and sensory/cognitive awareness [6]. The study, however, did not measure the long-term outcomes of HBOT in children with autism; additional studies are needed for this. Another randomized, double-blind placebo-controlled trial concluded that HBOT does not result in clinically significant improvement of the symptoms of ASD [7].

Gastrointestinal Therapy

Diet – gluten free / casein free diet (GF/CF): It has been suggested that peptides from gluten and casein may play a role in the origins of autism; hence the elimination of these peptides may improve behavior in children with ASD. The GF/CF diet is a commonly used treatment for autism. The rationale for this is based on the assumption that children with ASD have gastrointestinal problems including a “leaky gut.” The peptide fragments caused by the breakdown of casein and gluten are casomorphins and gliadinomorphins, respectively. It is believed that these by-products act centrally as endogenous opioids. The Cochrane Collaboration did a review of GF/CF diets for ASD. However, only one trial fit the inclusion criteria and was included. It was therefore concluded that there was not sufficient evidence to advise the GF/CF diet in persons with ASDs. A double-blind trial conducted using 15 children with autism did not identify statistically significant findings [8]. However, some parents reported subjective differences.

Vitamin B6 / magnesium: Studies investigating the effects of vitamin B6 in improving the behavior of children with ASD have been reported for the last few years. The Cochrane Collaboration completed a study investigating the efficacy of combined vitamin B6-magnesium treatment in persons with ASDs [9]. They looked at three randomized, double-blinded placebo-controlled clinical trials completed between 1993 and 2002. The first trial using low dose vitamin B6 provided insufficient data to conduct an analysis. The second trial using high dose vitamin B6 did not demonstrate any difference in improving symptoms such as communication, social interaction, impulsivity, compulsivity or hyperactivity in the treatment and placebo group [9]. Another study reported improvement in IQ and social quotient in children treated with B6 and magnesium [9]. All three studies suffered from methodological deficits and small sample size. There is therefore not sufficient evidence to demonstrate treatment efficacy.

Vitamin C: The use of vitamin C in the treatment of ASD has not been a popular treatment. A pilot study done to determine the effect of a moderate dose multivitamin/mineral supplement for children with autistic spectrum disorder concluded that there were low levels of vitamin C in children with autism and that high-dose supplementation led to clinical improvements [10]. The study, however, had many limitations.

Omega-3 fatty acids: There is increasing evidence that a lack or imbalance of polyunsaturated fatty acids such as omega-3 fatty acids may contribute to neurodevelopmental problems. Three studies found that omega-3 fatty acids were low in children with autism [11]. Numerous studies have been conducted to determine the safety and efficacy of omega-3 fatty acids for ASDs. Six studies that were done satisfied all inclusion criteria. Three other studies reported improvements in language and learning skills, clinical observations of anxiety, a clinician-administered symptom scale, and parental observations of general behavior and health [11]. An open-label study done concluded that there were no significant improvements observed in using omega-3 fatty acid supplementation in young adults with severe autism [12]. Because five studies were significantly limited by the lack of a control group and the presence of only one small randomized control trial, there is presently insufficient scientific evidence to determine if omega-3 fatty acids are effective in the treatment of ASDs.

Probiotics and antifungals: Overgrowth of the fungi Candida in the intestine was hypothesized to be a cause of symptoms of autism due to an underlying immune alteration, antibiotic use, and the use of processed sugars, which increase the growth of yeasts. The use of antifungals as treatment was based on a report of presumed candidal overgrowth in two boys who had autistic behavior, regression of milestones, and intermittent ataxia. A possible explanation for this was abnormal absorption because of the effect of yeast on the intestinal membrane and toxic effects of the yeast metabolites. No further cases or studies are available. Probiotic agents such as lactobacillus and acidophilus have been used as treatments for autism based on the hypothesis that there is an imbalance in intestinal microbes in individuals with ASD. There is insufficient information about the efficacy to make recommendations for their use. No clinical trials to date have examined these treatments for ASD.

Dimethylglycine (DMG): DMG is a derivative of the amino acid glycine. The use of DMG as treatment was based on the hypotheses that it was thought to reduce the lactic acid build up during stress, reduce seizure activity, and enhance oxygen use during times of hypoxia [13]. Two double-blinded placebo-controlled studies did not demonstrate differences between DMG and the placebos [13]. There is insufficient information about the efficacy and safety to make recommendations about its use in children with ASD.

Secretin: Secretin, a gastrointestinal (GI) hormone, has been extensively studied as a pharmacotherapeutic agent in the treatment of autism. A two-part clinical trial using 56 patients reported significant improvements in language function, social interaction, and GI symptoms. Some of these patients were later involved in a double-blind trial [14]. The study concluded that although there were transient changes in speech and behavior, it produced few meaningful changes when compared to the placebo group [14]. Various double-blind placebo-controlled trials have been done that concluded that the substance was no more useful than a placebo [15].

*Intranasal Oxytocin Therapy* 

In the last few years there have been many clinical trials on intranasal oxytocin. Few completed trials showed modest improvement in social function in ASD patients, but overall these trials showed inconclusive results. A double-blind, placebo-controlled trial in Australian University showed no benefit following oxytocin treatment. In this trial, 50 autistic male participants aged between 12 and 18 years were randomized to receive oxytocin or placebos. The primary outcomes were social responsiveness by caregivers and clinician-ratings. This trial showed no benefit and no side effects of oxytocin over placebos [16].

Non-biological therapies with no proven benefits

Auditory Integration Training (AIT)

The prevalence of sensory processing abnormalities in autism is relatively high. Language disorders in autism are often complicated by auditory problems such as hyperacusis. AIT involves listening to filtered, modulated music that presents sounds of varying volumes and pitches. It is typically administered in two daily half-hour sessions for approximately 10 days. The use of AIT in ASD is based on the theory that the continuous exposure to altered sound via headphones can functionally modify central auditory processing thus impacting language and behavior. Six randomized control trials were done, which were relevant [17-18]. Three of these studies reported improvement in overall autistic behavior [17]. Three did not show any benefit [18]. Scientific research has cast doubts on claims made for this as treatment of autism. The American Academy of Pediatrics has suggested that currently available information does not support the claims that AIT is an efficacious treatment and that their use does not appear warranted, except within research protocols.

*Sensory Integration Therapy (SIT)* 

Unusual sensory responses are said to be common in children with ASD. Sensory integration therapy is intended to focus directly on the neurological processing of sensory information as a foundation for learning of higher-level skills. The goal is not to teach specific skills or behaviors but to allow the child to interact with the environment more adaptively by correcting fundamental sensory-motor dysfunctions underlying the disorder. The treatment involves engaging the child in full body movements that are designed to provide tactile, proprioceptive, gravitational, auditory, visual, and vestibular stimulation. The treatment is typically provided by occupational therapists. Two single case studies comparing SIT with no-treatment baseline among children with autism have shown beneficial effects [19]. The studies, however, cannot determine if the benefits were specifically produced by SIT. The efficacy of sensory integration therapy has not been demonstrated objectively in studies done during the last eight years.

*Holding Therapy* 

Holding therapy has been proposed as a treatment for numerous childhood problems, including autism, since the 80s. The theory behind its use is that autism results from a lack of appropriate attachment of the child to mother. This defective bonding causes the child to withdraw, resulting in social and communicative deficits; therefore, if the mother provides the necessary physical contact required, the previously defective bond can be reestablished. The procedure is based on psychoanalytical theories of autism. No researchers have examined its efficacy.

Facilitated Communication

Facilitated communication is a technique where a trained facilitator physically guides the hand of a nonverbal child using an output device such as a keyboard, typewriter or similar device to spell. The use of facilitated communication in the treatment of ASD is based on the theory that persons with autism are nonverbal and that this technique allows individuals to overcome this condition and communicate appropriately. The procedure was initially heralded as a breakthrough in permitting communication between a parent and a previously uncommunicative child, and it, therefore, inspired great hope among family members especially parents of people with autism. However controlled studies demonstrated that if the facilitator guides the individual, then it is the facilitator and not the child that is the source of the typed information [20]. It has been officially refuted as a treatment modality by the American Academy of Pediatrics, American Academy of Child and Adolescent Psychiatry, American Psychological Association, and American Academy of Speech and Hearing.

Horseback Riding Therapy

The use of therapeutic horseback riding (hippotherapy) for children with ASD is based on the hypotheses that riding stimulates multiple domains of functioning such as social, cognitive, and gross motor [21]. In a nonrandomized study conducted, children with autism demonstrated improvements in distractibility, attention, and social motivation compared with controls [21]. The study had many limitations; therefore, further studies are necessary before this therapy can be recommended. It is important to note that horseback riding is associated with a risk of injury, so supervision is required at all times.

Dolphin-assisted Therapy (DAT)

Dolphin-assisted therapy has become an increasingly popular approach for intervention in children with disabilities including ASD. Dolphin-human interaction has been studied since the 1960s, and it has since been suggested that dolphins could help humans learn to communicate better with one another. The process involves swimming and interacting with dolphins typically in captivity [22]. Two peer-reviewed DAT studies that included children with autism were also methodologically flawed and plagued by several threats to both internal and construct validity. Two reviews of DAT concluded that there is no credible scientific evidence for the effectiveness of this intervention [22]. Science Daily published an article in December 2007 stating “Dolphin 'Therapy' A Dangerous Fad, Researchers Warn”.

Transcranial Magnetic Stimulation (TMS)

Transcranial magnetic stimulation is an energy-based therapy that works through electromagnetic induction to stimulate nerve cells in the brain. The use of TMS in the treatment of ASDs is based on the hypothesis that autism is related to a disturbance in cortical modularity [23]. A study conducted revealed that there was an association between reductions in repetitive-ritualistic behavior in treatment subjects [23]. The study, however, had many limitations. Further studies are needed to determine the efficacy of TMS in children with autism.

Music Therapy

People with autism have impairments in social interaction and communication. The theory behind using music therapy is that certain processes that occur in musical improvisation may help people with ASD to develop their capacity for social interaction and communicative skills. Music therapy is usually provided as individual therapy for people with ASDs. A meta-analysis conducted (Cochrane Collaboration) of three small studies concluded that music therapy might help to improve the communicative skills of children with ASD. However, only the short-term effects were examined and therefore it is still unknown how enduring the effects are on verbal and non-verbal communicative skills. The effects on behavioral problems were not significant. Another randomized controlled study concluded that there was supporting evidence in music therapy promoting social, emotional, and motivational developments in children with autism [24]. However a small sample size was used in this study and the test power was low. Both studies should, therefore, be interpreted with caution.

Biological therapies with proven benefits

*Psychopharmacologic Agent*s

No single medication has been proven to “treat” autism; rather there are medications that alleviate the signs and symptoms associated with autism. Psychopharmacologic agents are a useful adjunct to environmental and behavioral interventions in children with ASD. A trial of medications may be considered if symptoms cause significant impairment in functioning but only after treating any medical causes, initiating educational and behavioral interventions, and modifying any environmental factors. Pharmacological interventions may be considered for maladaptive behaviors such as self-injurious behavior, aggression, repetitive behaviors, mood lability, sleep disturbance, anxiety, hyperactivity, irritability, inattention, destructive behavior or other disruptive behaviors. When considering the use of medications for target symptoms, the potential benefits and risks must be weighed on a case-by-case basis.

Risperidone (atypical antipsychotic): Risperidone is the most commonly used antipsychotic for the treatment of disruptive behaviors in children with ASD. It was the first drug to be approved by the US Food and Drug Administration (FDA) for the symptomatic treatment of irritability, deliberate self-injury, and aggressive behavior in children (> five years old) with ASD. The use of risperidone for treating symptoms of hyperactivity and disruptive behaviors in children is supported by randomized controlled trials [25]. Two open-label studies have suggested tolerance and long-term benefits in using the drug [26]. Weight gain was a significant side effect of the drug [25].

Aripiprazole (atypical antipsychotic): Aripiprazole has also been approved by the FDA for the treatment of irritability in autistic children aged 6-17 years. Two double-blinded randomized placebo-controlled trials were conducted using the drug for eight weeks [27]. The results demonstrated reductions in stereotypy, irritability, and hyperactivity in children aged 6-17 years with autism as measured by the Aberrant Behavior Checklist and Clinical Global Impressions-Severity scale [27]. Adverse effects included vomiting, somnolence, weight gain, and fatigue [27]. The drug was, however, generally safe and well tolerated [27].

Potential Psychopharmacologic Agent Therapies That Require More Research

Stimulants: Recent double-blind placebo-controlled studies indicate that stimulants such as methylphenidate improves symptoms of hyperactivity, impulsivity, and inattention in children with ASD [28]. However, the response rate is lower in children with ASD than it is in children with isolated attention deficit hyperactivity disorder and side effects are more frequent. The side effects of the drug include—but are not limited to—decreased appetite, tics, irritability, jitteriness, abdominal discomfort, increased blood pressure and heart rate. Studies of amphetamines in the treatment of attention symptoms in children with ASD are lacking.

Alpha-adrenergic agonists: Alpha-2 adrenergic agonists such as guanfacine and clonidine have been used to manage symptoms of hyperactivity, impulsivity, and inattention in children with ASD. Two small double-blind placebo-controlled studies using clonidine suggested that there were some improvements in reducing hyperactivity, irritability, stereotypy, outbursts, and impulsivity [29]. However, further studies are needed in a larger autistic population to determine the dose-response relationship [29]. Adverse effects included sedation and hypotension. Guanfacine was also shown to be moderately effective in reducing hyperactive symptoms.

Serotonin-specific reuptake inhibitors (SSRIs): Various SSRIs have been used in the treatment of target symptoms of ASD. A randomized placebo-controlled crossover study using fluoxetine in children with ASD showed beneficial results in reducing repetitive and other maladaptive behaviors [30]. A double-blind two-way crossover trial using the drug concluded that it was effective in the treatment of young children with autistic disorder [31]. A study done to review literature about the use of SSRIs in the management of functional impairments associated with autistic disorder concluded that benefits in using SSRIs to treat the functional impairments in autism had been seen [32]. However the response to therapy and adverse effects are individualized and at present evidence does not support selection of one SSRI over another for any impairment associated with autism [32].

Nonbiological therapies with proven benefits

Applied Behavioral Analysis (ABA)

Programs based on ABA, which is an intensive behavioral intervention, are currently one of the most popular interventions for autism. ABA is a method of teaching appropriate behaviors by breaking down the tasks into small discrete steps and training in a systematic and precise way. It is characterized by a discrete presentation of stimuli with responses followed by immediate feedback, intense reinforcement, data collection, and systematic trials of instruction. ABA is used to reduce any interfering maladaptive behavior, to increase and reinforce desirable adaptive behaviors, teach new skills, and generalize behaviors to new situations and environments. The theory behind using ABA is that children with autism have difficulty in learning through imitation and listening as their normal peers do. The highly structured format seems to meet the needs of those who have autism and who typically respond to directness and routine. The techniques can be used in different types of situations such as natural everyday situations (e.g., during mealtime at home or play), structured situations (e.g., formal instruction in a classroom setting), and in 1-to-1 as well as group instruction setting. Examples of different types of ABA include the following:

Discrete trial training (DTT): This involves a type of teaching that uses a trial which consists of five parts 1) cue, 2) prompt, 3) response, 4) consequence, 5) inter-trial interval to teach each step of a desired response or behavior. The lessons learned are broken down into their simplest parts with positive reinforcement being used to reward a correct response or behavior. Incorrect responses are ignored.

Early intensive behavioral intervention (EIBI): This type of intervention is usually used for children with autism who are younger than 3-5 years of age.

Verbal behavioral intervention (VBI): VBI focuses on teaching verbal skills.

Pivotal response training (PRT): PRT targets areas such as motivation, responsivity to multiple cues, self-management, and social initiations. Any positive changes in behavior should have widespread effects on other behaviors.

The original study of ABA was conducted in 1987 and concluded that children who received ABA achieved significant gains in IQ scores [33]. Since then there have been numerous studies investigating the efficacy of ABA in children with autistic disorders. More recent studies have supported the use of ABA in autistic children. In 2009 a study was conducted on the comprehensive synthesis of EIBI for young children with autism based on the UCLA Young Autism Project model. The findings suggested that EIBI is an effective treatment on average, for children with autism [34]. It should be noted, however, that there were limitations in the study, which proves the need for further research. A review article concluded that there is a significant amount of scientific evidence in support of ABA treatment modalities for children with autism and it is therefore recommended for use. Meta-analytical methods for 22 studies suggested that long-term comprehensive ABA leads to (positive) medium-to-large effects regarding intellectual functioning, language development, acquisition of daily living skills and social functioning in children with autism [35]. Language-related outcomes were the most superior compared to other outcomes.

The National Autism Center’s National Standards Report considers intensive behavioral intervention to be an “established” treatment. It has been endorsed by some state and federal agencies, including the US Surgeon General and the New York State Department of Health.

A research paper in 2012 reviewed one randomized controlled trial (RCT) and four controlled clinical trials (CCTs) and concluded that there is some evidence that EIBI is an effective therapy [36]. But the same authors updated their paper in 2018 with recent RCTs and CCTs, which show that there is only weak evidence supporting EIBI [37].

Nonbiological therapies with possible benefits

TEACCH (Treatment and Education of Autistic and Related Communication-handicapped Children) 

This is a method of structured teaching that was established in the 1970s by Schopler and colleagues. It includes important elements such as the organization of the physical environment, predictable sequence of activities, routines with flexibility, structured work/activity systems, and visually structured activities [38]. It also provides clinical services such as social play and recreation groups, parent support groups and training, diagnostic evaluations, individual training for high functioning autistic groups, and supported employment (autismspeaks.org). The children work in a highly structured environment and are assessed to identify any new skills, and work then focuses on enhancing them. The programs are conducted in a classroom setting or may be home-based. Two studies that were done comparing TEACCH interventions with public education interventions found significant differences in scores on Psychoeducational Profile-Revised (PEP-R) on follow-up testing [39]. Another study in 2009 concluded that the effectiveness of TEACCH was confirmed showing positive outcomes in the natural settings and revealing its inclusive value [40].

Developmental Models

Developmental models focus on teaching skills that are essential to the development of a child, such as emotional relationships, social communication, and cognitive abilities. They are based on developmental theory and then organizing a hypothesis with regard to the fundamental nature of ASDs and then addressing the deficit with the relevant approach [38].

Denver model: The Denver model uses interpersonal relationships, play and activities to remediate the main deficits in imitation, emotion sharing, the theory of mind and social perception in order to foster symbolic thought and teach the power of communication [38].

Early Start Denver model (ESDM): ESDM is an integrative program that utilizes a combination of relationship-based and developmental approaches plus ABA programs. It includes parents as therapists.

Several studies by Rogers et al. have shown improvements in motor, play, cognitive, and social skills in children who have been treated according to the Denver model. A randomized control trial of 48 children with ASD evaluating the efficacy of the ESDM concluded that there were significant improvements in adaptive behavior, language and IQ, and autism diagnosis compared to children who received only community intervention [41]. It was also noted that the ESDM group maintained its rate of growth in adaptive behavior and were also more likely to experience a change to pervasive developmental disorder, not otherwise specified as compared to the community intervention group [41]. Follow-up studies will be required to investigate whether the ESDM group would sustain their gains over a long term.

Developmental individual difference (DIR): DIR was developed in the 1980s by Dr. Stanley Greenspan. It focuses on “floor time” play sessions and other strategies that enhance relationships and emotional and social interactions in order to facilitate cognitive and emotional development. It also addresses deficits in motor planning and sequencing, auditory processing and language, visual spacing processing, and sensory modulation [38]. The parent or therapist engages the child at a level the child currently enjoys, enters the child’s activities, and follows the child’s lead. Further studies are needed to investigate the efficacy of DIR in children with autism.

Relationship developmental intervention (RDI): RDI focuses on doing activities that elicit interactive behavior that encourages the child to engage in a social relationship. This, in turn, allows the child to discover the value of positive interpersonal activity and thus helps him or her to become more motivated in learning the skills to sustain this relationship [38]. The children work in a one-to-one setting with a parent who has been trained in the process. A study evaluating the RDI program concluded that there were reductions in autistic symptoms and increased mainstream placements in an uncontrolled study of 16 children who received the intervention [42].

Responsive Teaching (RT)

Responsive teaching is an early intervention curriculum implemented by parents in order to address the language, cognitive, and social-emotional needs of young children with developmental problems. A study in 2005 reported significant improvements in cognition, communication, and social functioning in children with pervasive developmental disorders and developmental disabilities [43]. Parents were taught to use RT strategies to encourage children to acquire and use pivotal developmental behaviors. The children’s improvements were related to their own pivotal behavior and their parent’s responsiveness.

Picture Exchange Communication System (PECS)

Picture exchange communication system is an augmentative communication system frequently used in children with autism. Its primary purpose is to teach children with autism to initiate communication by handing a picture to a communication partner in exchange for the desired item. The picture may be used instead of or in conjugation with speech. A randomized control trial of 84 participants showed the modest effectiveness of PECS teacher training/consultancy [44]. The use of symbols in the classroom and the rates of the children’s initiations increased [44]. There was no evidence of improvement in other areas of communication and the effects were not maintained once active intervention stopped. Another study investigated the effects of PECS teaching to phase 3 on the communicative interactions between children with autism and their teachers. There were significant increases in communicative initiations and dyadic interactions between the children and teachers in the PECS group as compared to the control group [45].

Pivotal Response Training (PRT)

Pivotal response training is a behavioral intervention model based on the principles of ABA. The intervention focuses on the “pivotal” behaviors such as motivation and initiation of communication with others that in turn affects a wide range of other behaviors. The goal is to produce positive changes in the “pivotal” behaviors, which in turn leads to an improvement in social skills, play skills, and communication skills and allows the child to monitor his own behavior. PRT is also used to decrease self-stimulatory and disruptive behavior. It is usually provided by specially trained staff.

A study on the efficacy of PRT found that it was effective in producing positive changes in the social behavior of children with autism. Initiations increased with all peer trainers [42]. Due to its small sample size, the study should be interpreted with caution.

Caregiver-mediated Intervention

In this method caregivers actively coached the children everyday activities like watering plants, grooming, and helping with laundry. The intervention followed the JASPER (Joint attention symbolic play engagement and regulation) treatment [46]. A multisite, randomized, comparative study based on this method was conducted in 2014 on a significant number of low-resourced children who have ASD. In this study, 112 families were randomly assigned for intervention and the children were assessed for pre- and post-treatment social communication skills and followed up to three months [46]. The results showed significant improvement in outcomes of joint engagement and joint attention, symbolic play. The limitation for this type of intervention is that without active support to caregivers there is a difficulty to continue the interventions [46].

Parent-mediated Communication

In this method, parents are trained by the therapist with the aim to first increase parental sensitivity and responsiveness to child communication and reduce mistimed parental responses. A randomized control trial using the preschool autism communication trial (PACT), published in the Lancet, 2010, tests this method. This study recruited 152 children, of whom 77 were assigned the PACT method and 75 were assigned the usual methods of treatments. Autism symptoms improved in both groups and only marginally more in the PACT intervention group; so they concluded that they do not recommend this method to treat autism [47].

After six years, in 2016, they repeated the study again using the PACT method, and they observed a significant reduction in long-term outcomes and a significant reduction in symptom severity scores (effect size 0.55; 95% CI 0.14, p=0.004) [48]. This is the first study showing convincing benefits for autism patients, and this intervention mediated by parents was relatively inexpensive [48].

In 2015, two types of parent-mediated communication methods were tested in an RCT [49]: 1) a naturalistic, developmental, behavioral intervention (joint attention, symbolic play, engagement, and regulation-JASPER); 2) a parent-only psychoeducational intervention (PEI). In this RCT, 86 toddlers (range 22-36 months) with ASD participated, and results showed that the JASPER method had a significant effect on joint engagement comparatively over the PEI method [49].

Social ABCs by parents or caregivers [50]: This method consisting of a 12-week parent-mediated intervention was tested in 2017 in a randomized control trial on 63 autistic children in Canada. The results showed that using this method improved children's vocal responsiveness, children’s initiation to their caregivers, and social communication. The treatment group experienced a favorable response in these features: (1) child functional vocal responsiveness to parent prompts (R2 = 0.43, P < .001), (2) child vocal initiations (R2 = 0.28, P < .001), (3) parent smiling (R2 = 0.09, P = .017), and (4) fidelity of implementation (R2 = 0.71, P < .001) [50]. But this method showed improvement in only two skills (vocal responsiveness and parental positive effect), and hence this method needs more research.

## Conclusions

Autism spectrum disorder is a pervasive developmental disorder with a multifactorial etiology affecting 1/110 children worldwide. It continues to remain a challenging condition for children and their families; however, significant advances have been made regarding diagnosis and management. It is important to realize that the etiology of autism is unknown and at present, there is no cure, although there are interventions that may be effective in alleviating some symptoms and improving skills that may help autistic persons lead more productive lives. Because of the substantial impairments in autistic children, family members desperately turn to complementary and alternative interventions that may or may not have been proven to work. It is extremely important as health care professionals to be educated about the complementary, alternative, and evidence-based interventions available, so that the families of autistic children can obtain the best and effective treatment for their children.
